# Association between focal amyloid deposition and cognitive impairment in individuals below the amyloid threshold

**DOI:** 10.3389/fnagi.2024.1452081

**Published:** 2024-10-30

**Authors:** Hongki Ham, Byeong C. Kim, Eun Hye Lee, Daeun Shin, Hyemin Jang, Sung Hoon Kang, Jihwan Yun, Hee Jin Kim, Duk L. Na, Jun Pyo Kim, Sang Won Seo, Soo Hyun Cho

**Affiliations:** ^1^Department of Neurology, Samsung Medical Center, Sungkyunkwan University School of Medicine, Seoul, Republic of Korea; ^2^Neuroscience Center, Samsung Medical Center, Seoul, Republic of Korea; ^3^Alzheimer's Disease Convergence Research Center, Samsung Medical Center, Seoul, Republic of Korea; ^4^Department of Neurology, Chonnam National University Hospital, Chonnam National University Medical School, Gwangju, Republic of Korea; ^5^Department of Neurology, Seoul National University Hospital, Seoul National University College of Medicine, Seoul, Republic of Korea; ^6^Department of Neurology, Korea University Guro Hospital, Korea University College of Medicine, Seoul, Republic of Korea; ^7^Department of Neurology, Soonchunhyang University Bucheon Hospital, Bucheon-si, Gyeonggi-do, Republic of Korea; ^8^Department of Digital Health, SAIHST, Sungkyunkwan University, Seoul, Republic of Korea; ^9^Department of Health Sciences and Technology, SAIHST, Sungkyunkwan University, Seoul, Republic of Korea; ^10^Department of Intelligent Precision Healthcare Convergence, Sungkyunkwan University, Suwon, Republic of Korea; ^11^Happymind Clinic, Seoul, Republic of Korea

**Keywords:** amyloid PET, centiloid, focal amyloid deposition, neuropsychological characteristics, structural changes

## Abstract

**Purpose:**

This study aimed to investigate the characteristics of individuals with amyloid levels below the threshold. To achieve this, we differentiated between two groups: those with global amyloid negativity but focal deposition [G(–)F(+)] and those without focal deposition [G(–)F(–)].

**Materials and methods:**

A total of 2,677 participants were diagnosed with cognitive unimpairment (CU) or mild cognitive impairment (MCI). MRI-based regional centiloid (CL) values were used to establish threshold values for each brain region. After applying a cutoff of 20 rdcCL to identify amyloid positivity, participants who were globally amyloid-negative were grouped into three categories: those who showed focal amyloid uptake [G(–)F(+)], individuals without focal amyloid deposition but with relatively high CL(HC) levels comparable to those in the focal uptake group [G(–)F(–) HC)], and those with relatively low CL(LC) levels [G(–)F(–) LC]. We compared the neuropsychological test results and brain structural changes between these groups using ANCOVA.

**Results:**

The G(–)F(+) group demonstrated a lower cortical thickness (*P* < 0.001) than the G(–)F(–) HC group. In neuropsychological tests, the G(–)F(+) group exhibited lower the Seoul Verbal Learning Test delayed recall (SVLT-DR) and Mini Mental State Examination (MMSE), and showed progressed clinical status in the clinical dementia rating–sum of boxes (CDR-SOB) compared to the G(–)F(–) HC group (*P* < 0.001). The subsequent sensitivity analyses confirmed the persistence of these findings.

**Conclusions:**

Individuals with focal amyloid deposition [G(–)F(+)] exhibited higher rates of cognitive impairment compared to patients with similar levels of amyloid, underscoring the importance of monitoring the progression of focal uptake, even when it remains below the amyloid threshold.

## 1 Introduction

Detection of amyloid deposition in patients can be considered an early sign of dementia. In the past, this feature could not be confirmed *in vivo*; However, in 2004, a method was introduced to measure amyloid deposition as a diagnostic and predictive biomarker of Alzheimer's disease (AD). This method utilizes Pittsburgh compound B (PiB), which contains the carbon isotope ^11^C (Klunk et al., 2004). PiB has a high affinity for fibrillar amyloids in amyloid aggregates, making it possible to determine the extent of amyloid deposition. Subsequently, flutemetamol (FMM) and florbetaben (FBB), which utilize similarly acting fluorine isotopes which are not limited by a short half-life like PiB, were developed and imaged using positron emission tomography (PET) with ligands (Zhang et al., 2005; Rowe et al., 2016, 2017; Battle et al., 2018; Navitsky et al., 2018). As various other ligands have been developed, it has become necessary to unify different imaging values into one score; to solve this problem, a centiloid (CL) was developed. The CL values were standardized by scaling the PiB values of young controls and participants with typical AD dementia from 0 to 100 (Klunk et al., 2015). This conversion formula can also be used to calculate the CL of ligands such as FMM and FBB. However, because of the limitations of PiB, CL has become difficult to use in many medical institutions; as such, a direct comparison of CL (dcCL) has been developed to allow the direct comparison of FMM and FBB (Cho et al., 2020a). Since then, to solve the disadvantage of inaccurate regional uptake, research on the use of rdcCL to observe regional uptake has been conducted.

In prior longitudinal studies (Farrell et al., 2018; Leal et al., 2018), individuals who were initially cognitively unimpaired (CU), tested negative for amyloid, and had PiB values below the threshold at the onset of the study showed a slight increase in tau accumulation and a decline in memory performance as their PiB values increased. In one study, the correlation between memory impairment and amyloid levels remained unclear below the threshold; however, in amyloid-positive patients, there was a correlation between memory decline and amyloid levels. This indicates that amyloid-induced tau accumulation can occur even when amyloid is negative and that tau accumulation can lead to memory decline (Leal et al., 2018). Another study reported that even if patients are amyloid-negative, focal accumulation of amyloid can be linked to memory decline (Farrell et al., 2018).

Two other studies consistently identified progression in structural changes and cognitive decline in groups with focal deposition. One study compared patients with focal uptake to those without focal uptake among globally negative patients (Kim S. E. et al., 2021). Another study developed a computed tomography (CT)-based regional modified CL to better detect these changes (Kim et al., 2022). Additionally, a longitudinal study using FMM noted that patients with focal uptake experienced more pronounced disease progression over time than those without focal uptake.

In this context, the present study aimed to determine the causes of cognitive impairment in patients with amyloid levels below the threshold level. Possible causes may include relatively high amyloid levels despite being negative for amyloid, as seen in the first cited study (Leal et al., 2018), or local amyloid deposition in certain brain regions, as observed in the second cited study (Farrell et al., 2018), in which amyloid was negative in the entire brain, but locally accumulated in certain regions, potentially leading to cognitive impairment.

To investigate these hypotheses, the following analyses were conducted: First, magnetic resonance imaging (MRI)-based regional CL values were used to determine the rdcCL threshold values for each region. Second, patients who showed focal amyloid deposition despite an overall negative rdcCL value, and those who had a similar rdcCL level but no focal deposition, were stratified. Finally, the neuropsychological characteristics of these patient groups were compared with brain structural changes such as cortical thickness and hippocampal volume.

## 2 Methods

### 2.1 Subjects

We recruited a total of 2,667 participants who were either cognitively unimpaired (CU) (*n* = 1,020) or had a mild cognitive impairment (MCI) (*n* = 1,647) and underwent an Aβ PET scan at the Samsung Medical Center between August 2015 and March 31, 2023. The CU participants were composed of spouses of patients who visited the memory clinic, volunteers who applied for comprehensive dementia evaluation advertised in the paper, and participants who had cognitive complaints. The diagnostic criteria for CU were as follows: (1) no medical history that could potentially affect cognitive function based on Christensen's health screening criteria (Christensen et al., 1991) (2) no objective cognitive impairment in any cognitive domain, as determined by a comprehensive neuropsychological test battery [performance above at least −1.0 standard deviation (SD) of age-adjusted norms on any cognitive test]; and (3) independence in activities of daily living. The criteria for diagnosing MCI were based on the 2011 National Institute on Aging-Alzheimer's Association Diagnostic Guidelines (Albert et al., 2011; Mckhann et al., 2011; Petersen et al., 1999).

Clinical interviews, neuropsychological assessments, complete blood count, blood tests, thyroid function tests, and laboratory examinations were performed. Structural abnormalities, such as cerebral infarction, brain tumors, and vascular deformities, were ruled out through MRI. The study was approved by the Institutional Review Board (IRB) of the SMC, and written informed consent was obtained from all participants.

### 2.2 Brain MRI acquisition

A 3.0-T MRI scanner (Philips Achieva; Philips Healthcare, Andover, MA, USA) was used with the following parameters: the sagittal plane was scanned with a thickness of 1.0 mm, without any gap between adjacent sections, with half of the slices being duplicated; the repetition time was set to 9.9 ms, and the echo time was 4.6 ms; the flip angle was set to 8°; the matrix size was 240 × 240 pixels and was reconstructed into 480 × 480 pixels from a 240 mm imaging area.

The CIVET pipeline (version 2.1.0) developed at the McConnell Brain Imaging Centre (BIC) was employed for structural analysis. The participants' MRI images were linearly transformed to the MNI-152 template and corrected for intensity non-uniformity using the N3 algorithm. The resulting images were segmented into white matter, gray matter, cerebrospinal fluid, and non-brain regions. Subsequently, hemispherical separation was performed to extract the surfaces (Collins et al., 1994; Sled et al., 1998; Zijdenbos et al., 2002).

As cortical thickness is associated with the total intracranial volume (ICV), ICV was estimated according to previous studies (Kang et al., 2019). ICV was defined as the total volume of gray matter, white matter, and cerebrospinal fluid, and the Brain Extraction Tool (BET) algorithm was applied to calculate the total voxel count of the brain mask in the MRI image and measure the volume (Smith, 2002). Cortical thickness values were computed in the native brain space instead of the Talairach space, because of the constraints imposed by linear stereotaxic normalization techniques. The cortical surface model extracted from the MRI image in the stereotaxic space was transformed back into the native space using inverse transformation matrices, followed by reconstruction of the cortical surface. The hippocampal volume was determined using a fully automated approach based on the graph-cut algorithm employed in previous studies (Kwak et al., 2013).

### 2.3 Amyloid PET imaging acquisition, analysis, and centiloid values

Positron emission tomography (PET) images of both FMM and FBB were obtained using a Discovery STE PET/CT scanner (GE Medical Systems, Milwaukee, WI, USA), utilizing a 3D scanning process to obtain 47 slices with a thickness of 3.3 mm. After injection of either FMM or FBB according to the ligand manufacturer's protocol, dynamic-mode PET scans were performed for 20–90 min after injection, comprising four frames of 5 min each. The 3D PET images were then reconstructed into 128 × 128 × 48 shapes with a voxel size of 2 × 2 mm × 3.27 mm using the ordered-subsets expectation maximization (OSEM) algorithm (Jang et al., 2019).

The standard method used in this study is based on the CL Project (Klunk et al., 2015). The obtained data were normalized using Statistical Parametric Mapping version 8 (SPM8). To create global and regional CL volumes of interest (VOIs), the entire cerebellum mask provided by The Global Alzheimer's Association Interactive Network (GAAIN) was used as the reference region. To define the area of amyloid accumulation common to both FMM and FBB PET images, images from 25 amyloid-positive and 18 amyloid-negative patients enrolled in a head-to-head cohort were used. For each participant, four average images were obtained for each ligand in both the positive and negative groups, and the positive-dominant FBB and FMM CTX VOIs were defined by subtracting the negative images from the positive images for each ligand. The top 20% of voxels with the strongest intensity among the overlapping regions of these VOIs were defined as the FBB-FMM CTX VOI (Tzourio-Mazoyer et al., 2002).

To calculate the MRI-based regional CL values, the process of obtaining standardized uptake value ratios (SUVR) for each patient from the normalized PET images using VOIs of the whole cerebellum mask and defined VOIs of five sub-regions (Barthel et al., 2011) (Frontal, Parietal, Posterior Cingulate, Temporal, and Striatum) based on the Automated Anatomical Labeling (AAL) atlas was further performed (Kim et al., 2022). These five regions we chose were based on recommendations from the Flutemetamol (FMM) guidelines for visual assessment. Although the Florbetaben (FBB) guidelines do not explicitly include the striatal region, we consider its inclusion crucial due to its potential relevance in amyloid deposition and cognitive impairment. The intensity of each sub-region was divided by the intensity obtained from the VOI of the entire cerebellum, which served as the reference region. These values represented the regional SUVR, while the sum of the five sub-region VOIs constituted the VOI for the entire cortex. Using the same method, the SUVR values for the entire cortex were obtained. SUVR values obtained for the entire cortex were transformed into rdcCL values using the CL conversion formula:


$$\begin{array}{l} CL\;=\;100^{*}\left(SUVr_{ind}-SUVr_{YC-0}\right)/\left(SUVr_{ADCI-100}-SUVr_{YC-0}\right) \end{array}$$

Where *SUVr*_*ind*_ refers to individual SUVR values, while *SUVr*_*ADCI*−100_ and *SUVr*_*YC*−0_ represent the average SUVR values of participants with AD and young healthy controls, respectively, corresponding to CL values of 100 and 0, indicating the extent of standardized uptake values. This process yielded a transformation equation for the SUVR values of the entire cortex into CL values, and a similar process was applied to obtain regional CL values by performing the same steps on the regional SUVR values. This study utilized BeauBrain Healthcare Morph's image processing technology to examine brain atrophy and classify Alzheimer's Disease using MR images.

### 2.4 Classification of participants using centiloid threshold

We calculated cutoff values for focal Aβ accumulation, using previously obtained regional volumes of interest (VOIs) and individually obtained global VOI areas which were found to be common areas of accumulation for both FBB and FMM, and were consistent with the recommended regions for visual interpretation in the FBB and FMM guidelines.

The threshold value was obtained using a Gaussian mixture model (GMM), an unsupervised learning algorithm that clusters data using a Gaussian distribution. After clustering, the highest number of patients in the low-CL group was used as a criterion to separate positive and negative cases. For the GMM clustering, we utilized the rdcCL data obtained from 3,753 participants aged 55 or above. All participants, including those with dementia of Alzheimer's type (DAT), were used to determine the threshold, whereas for the group comparison of structural and cognitive changes, we included only CU and MCI participants, and excluded DAT participants. Among the 3,753 participants, 2,176 had FMM and 1,577 had FBB. Finally, we determined the global cut-off value for rdcCL using the same methodology. However, owing to subsequent issues, the rdcCL threshold for the entire region was not based on the threshold obtained in the previous process. The threshold values in five regions, including the frontal, parietal, posterior cingulate, temporal, and striatal regions were obtained using a Gaussian mixture model (GMM). The threshold values were 26.91 in the frontal, 26.24 in the parietal, 29.22 in the posterior cingulate, 29.32 in the temporal, and 36.86 in the striatal regions.

Participants who did not exceed a rdcCL of 20 were classified as negative for the entire region, whereas those who exceeded the threshold were classified as positive. Among the participants classified as negative for the entire region [G(–)], we further distinguished between regional positives and negatives based on the regional CL threshold to define regional positives as the focal group [Global(-)Focal(+), G(–)F(+)]. To match the rdcCL levels of the G(–)F(+) group with those of the G(–)F(–) group, we chose to define the top 20% as our threshold for high centiloids (G(–)F(–) HC group). We also incorporated the G(–)F(–) Low Centiloid (LC) group in our study to serve as a comparison alongside the G(–)F(–) High Centiloid (HC) and G(–)F(+) groups.

**Figure 1:**
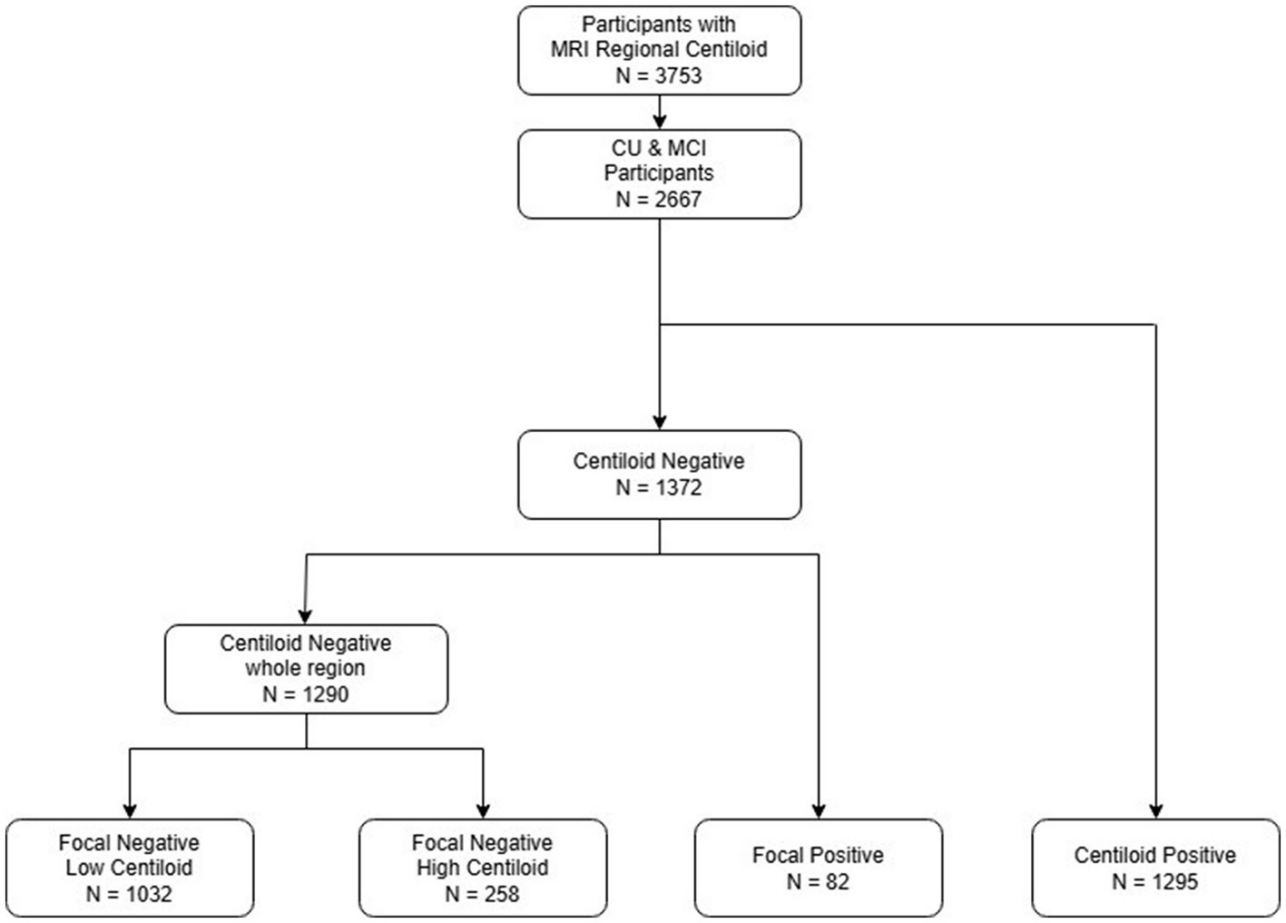
Flowchart of the study participants. An analysis of CU and MCI participants was conducted, and patients were categorized into four groups: focal negative with low centiloid, focal negative high centiloid, focal positive, and global positive. CU, cognitive unimpaired; MCI, mild cognitive impairment.

Figure 1 is a flowchart illustrating the process of dividing the 3,753 participants into three groups. We included 2,667 participants with CU and MCI, of whom 1,372 were overall negative and 1,295 were overall positive, based on the 20 rdcCL. The 1,290 participants who did not show focal uptake were subdivided into a low CL group [G(–)F(–) LC] (*n* = 1,032) and a high CL group [G(–)F(–) HC] (*n* = 258), and those who showed focal amyloid uptake in five areas were classified into a focal group [G(–)F(+)] (*n* = 82).

For the sensitivity analysis, we selected cutpoints that are either more conservative or more liberal than the initial cutpoint of 20 CL to ensure a comprehensive evaluation. Specifically, we calculated a cutpoint of 25.5 CL using GMM, which provides a robust statistical basis for this threshold. Additionally, we selected a cutpoint of 10 CL based on findings from previous literature, which indicated that CL values < 10 accurately reflected the absence of any neuritic plaque (Amadoru et al., 2020).

### 2.5 Neuropsychological evaluation

All participants completed the Seoul Neuropsychological Screening Battery (SNSB) (Ahn et al., 2010), including the Rey–Osterrieth Complex Figure Test delayed recall (RCFT-DR), Seoul Verbal Learning Test delayed recall (SVLT-DR), Mini Mental State Examination (MMSE) (Kim et al., 2024), and clinical dementia rating sum of boxes (CDR-SOB). Except for the CDR-SOB, the remaining neuropsychological features were analyzed following z-score standardization. Not all participants completed every test, and missing values for each test were considered in the analysis of the results.

### 2.6 Analysis of the regional distribution of focal amyloid deposition

For the G(–)F(+)group, we categorized participants based on whether they had amyloid deposition in a single region or multiple regions. Additionally, we analyzed the distribution of focal amyloid across these regions to explore any potential trends in cognitive or structural outcomes associated with specific deposition sites. Statistical comparisons were performed between participants with single-region uptake vs. those with multiple-region uptake, as well as across different regional subgroups. We also performed analyses to investigate whether the areas where localized amyloid deposition occurred had different effects than other areas.

### 2.7 Statistical analysis

Among the three groups other than global positive, we conducted chi-square tests to compare categorical data and analysis of covariance (ANCOVA) to analyze differences in neuropsychological test scores and structural measures between the groups. Covariates used for neuropsychological test scores included age, sex, APOE ε4, and education level, whereas age, sex, APOE ε4, and ICV were used for the analysis of structural differences. Among the three groups other than global positive, Bonferroni correction was applied for *post hoc* tests. In the case of global positivity, only comparison with focal positivity was conducted, and ANCOVA was conducted in the same manner, but *post-hoc* was not conducted. All statistical analyses and visualizations were performed using Python version 3.8.3. The scipy package was used for the chi-square test, the Pingouin package was used for ANCOVA and Bonferroni *post hoc* analysis, and the seaborn package and statannotate package were used for data visualization.

## 3 Results

### 3.1 Demographics of the participants

Table 1 summarizes the demographic characteristics of the four groups. Among the 2,667 participants, 1,032 (38.70 %) were stratified in the G(–)F(–) LC group, 258 (9.67%) in the G(–)F(–)HC group, 82 (3.07 %) in the G(–)F(+) group, and 1,290 (48.56%) in the G(+) group. There were no differences in centiloid values between the G(–)F(+) and G(–)F(–) HC groups (*P* = 1.000). Age was highest in the G(–)F(+) group, followed by the G(+) group, and lowest in the G(–)F(–)HC group (*P* < 0.001). When comparing APOE ε4 carrier status, the G(+) group had the highest proportion, followed by the G(–)F(+) group, the G(–)F(–) HC group, and finally the G(–)F(–) LC group. No differences were found between the groups regarding hypertension, hyperlipidemia, cardiac disease, or stroke. However, for diabetes mellitus, the G(+) group (18.1%) showed significantly lower compared to the G(–)F(–) LC group (22.4%, *P* = 0.011) and the G(–)F(–) HC group (24.0%, *P* = 0.032).

**Table 1:** Demographics of the participants by group.

**Group**	**Total**	**Global(–) Focal(–)**		**Global(–) Focal(+)**	**Global(+)**
		**Low Centiloid**	**High Centiloid**		
No. (%)	2,667 (100.0)	1,032 (38.70)	258 (9.67)	82 (3.07)	1,290 (48.56)
Global Centiloid, mean ±SD	44.9 ± 52.9	−2.1 ± 7.1	12.4 ± 3.1^*^	11.7 ± 7.0^*^	90.9 ± 39.4^*†‡^
Age, mean ± SD, year	71.97 ± 7.47	71.5 ± 7.7	69.5 ± 6.7^*^	74.9 ± 6.6^*†^	72.7 ± 7.3^*†‡^
Female Sex, No (%)	1,542 (57.8)	583 (56.5)	135 (52.3)	35 (42.7)^*^	789 (60.9)^*‡^
Education, mean ± SD, year	11.9 ± 4.7	12.1 ± 4.7	11.8 ± 4.9	12.9 ± 4.6	11.8 ± 4.7
Diagnosis (CU/MCI), No.	1,020/1,647	530/502	131/127	31/51^*^	328/967^*†‡^
APOE ε4 Carrier, No. (%)	966 (36.2)	125 (12.1)	49 (19.0)^*^	36 (43.9 )^*†^	756 (58.4)^*†‡^
Hypertension, No(%)	504 (48.8)	504 (48.8)	123 (47.7)	43 (52.4)	600 (46.3)
Diabetes mellitus, No(%)	544 (20.4)	231 (22.4)	62 (24.0)	17 (20.7)	234 (18.1)^*†^
Hyperlipidemia, No(%)	1198 (44.9)	452 (43.8)	113 (43.8)	45 (54.9)	588 (45.4)
Cardiac Disease, No(%)	155 (13.3)	156 (15.1)	28 (10.9)	12 (14.6)	159 (12.3)
Stroke, No(%)	119 (4.5)	50 (4.8)	11 (4.3)	6 (7.3)	52 (4.0)

Statistical analyses were performed with Chi-square tests for sex, *APOE* ε4 and diagnosis. Analysis of covariance (ANCOVA) was used to analyze age and education. After applying a cutoff of 20 rdcCL to identify amyloid positivity, participants who were globally amyloid negative grouped into three categories: those who showing focal amyloid uptake [G(–) F(+)], individuals without focal amyloid deposition but relatively high CL (HC) level comparable to the focal uptake group [G(–)F(–) HC], and those with relatively low CL (LC) levels [G(–)F(–) LC]. SD, standard deviation; CU, cognitive unimpaired; MCI, mild cognitive impairment. ^*^P < 0.05 group vs. G(–)F(–) LC. ^†^*P* < 0.05 group vs. G(–)F(–) HC. ^‡^*P* < 0.05 G(+) vs. G(–)F(+).

### 3.2 Comparisons of brain structural features among each group

**Figure 2:**
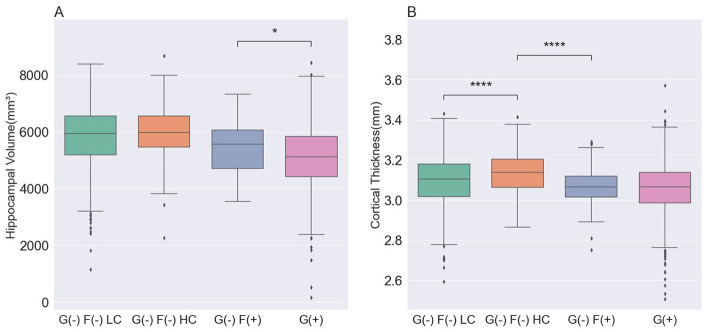
Comparison of the structural features in participants. **(A)** The hippocampal volume was lowest in the G(+) group, followed by the G(–)F(+), G(–)F(–) LC, and G(–) F(-) HC groups. **(B)** The cortical thickness was lowest in the G(+) group and G(–)F(+) group, followed by the G(–)F(-) LC, and G(–)F(-) HC groups. *P* values for differences between groups were calculated from an analysis of covariance with age, sex, *APOE* ε4 and ICV, followed by a Bonferroni *post hoc* test. G(–)F(–) LC, global negative and focal negative with low centiloid; G(–)F(–) HC, global negative and focal negative with high centiloid; G(–)F(+), global negative and focal positive; G(+) global positive. *, 0.01 < *P* ≤ 0.05; ****, *P* < 0.0001.

Brain structural features were assessed by measuring the hippocampal volume (HV) and global cortical thickness (Table 2, Figure 2). Among the G(–) group, The G(–)F(+) group showed the lowest results for both HV and cortical thickness. When comparing the three groups within the G(–) group, ANCOVA did not show differences in HV (*P* = 0.985). G(–)F(+) (3.07 ± 0.11 mm) showed significantly lower cortical thickness (*P* < 0.001) compared to G(–)F(–) HC (3.13 ± 0.10 mm). However, HV differed between the G(–)F(+) and G(+) groups (*P* = 0.002), while no differences were observed in global thickness (*P* = 0.314).

**Table 2:** Comparison of hippocampal volumes, cortical thickness and cognitive measures among the four groups.

	**Global(–) Focal(–)**		**Global(–) Focal(+)**	**Among the G(–) groups^§^**	**Global(+)**
	**Low Centiloid**	**High Centiloid**			
Hippocampal volumes (mm^3^)	5,834.65 ± 1,012.56	6,005.85 ± 871.32	5,431.76 ± 864.52	0.985	5,109.11 ± 1,027.33^‡^
Cortical Thickness (mm)	3.09 ± 0.12	3.13 ± 0.10^*^	3.07 ± 0.11^‡^	0.009	3.06 ± 0.13
SVLT-DR (z-scores)	−0.53 ± 1.27	−0.54 ± 1.27	−1.12 ± 1.25^*†^	0.009	−1.55 ± 1.30^‡^
RCFT-DR (z-scores)	−0.46 ± 1.15	−0.26 ± 1.17	−0.95 ± 1.11	0.660	−1.31 ± 1.12^‡^
MMSE Score (z-scores)	−0.48 ± 1.60	−0.33 ± 1.26	−1.05 ± 1.94^*†^	0.014	−1.76 ± 2.34^‡^
CDR-SOB	1.17 ± 1.34	1.02 ± 0.83	1.81 ± 2.09^*†^	< 0.001	2.20 ± 2.16

Values are presented as the mean ± standard deviation. We compared brain structural features and neuropsychological characteristics between the four groups. Statistical analyses were performed using analysis of covariance (ANCOVA). A *post hoc* test was performed using the Bonferroni correction. Covariates used for neuropsychological test scores included age, sex, APOE ε4 and education level, whereas age, sex, APOE ε4 and ICV were used for the analysis of structural differences. SVLT-DR, Seoul verbal learning test-delayed recall; RCFT-DR, Rey-Osterrieth complex figure test-delayed recall; MMSE, mini-mental state examination; CDR-SOB, clinical dementia rating scale sum of boxes; G(–) F(–), global negative and focal negative; G(–) F(+), global negative and focal positive; G(+) global positive. ^*^P < 0.05 group vs. G(–)F(–) LC among G(–) groups. ^†^*P* < 0.05 group vs. G(–)F(–) HC among G (–) groups. ^‡^*P* < 0.05 G(+) vs. G(–)F(+). ^§^Values are *P*-values of ANCOVA among the G(–) groups.

### 3.3 Comparison of neuropsychological characteristics in each group

**Figure 3:**
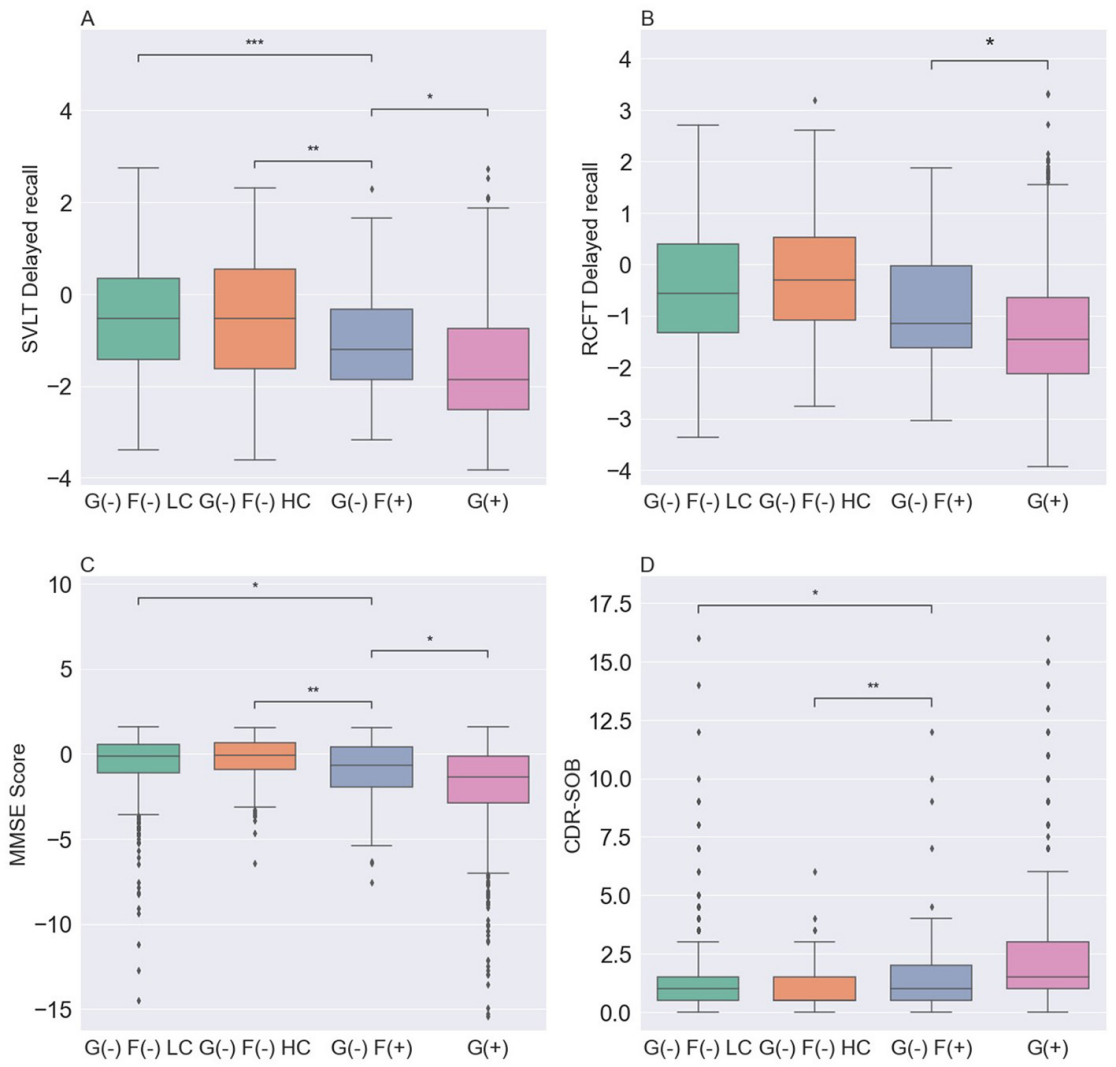
Comparison of neuropsychological characteristics among the four groups. **(A)** SVLT-DR was lowest in the G(+) group. **(B)** RCFT-DR was lowest in the G(+) group, followed by the G(–)F(+), G(–)F(–) LC and G(–)F(–) HC groups. **(C)** MMSE was lowest in the G(+) group. **(D)** CDR-SOB was highest in the G(+) group. *P* values for differences between groups were obtained by analysis of covariance for age, sex, *APOE* ε*4*, and education, followed by Bonferroni *post hoc* test. SVLT-DR, Seoul verbal learning test-delayed recall; MMSE, mini-mental state examination; CDR-SOB, clinical dementia rating scale sum of boxes; G(–)F(–) LC, global negative and focal negative with low centiloid; G(–)F(–) HC, global negative and focal negative with high centiloid; G(–) F(+), global negative and focal positive; G(+) global positive. *, 0.01 < *P* ≤ 0.05; **, 0.001 < *P* ≤ 0.01; ***, 0.0001 < *P* ≤ 0.001.

We compared the neuropsychological characteristics of each group using the SVLT-DR, RCFT-DR, MMSE, and CDR-SOB scores (Table 2, Figure 3). Among the three G(–) groups, the G(–)F(+) group performed lower than the G(–)F(–)HC and G(–)F(–)LC groups in the SVLT-DR, MMSE, and CDR-SOB scores except for the RCFT delayed recall (*P* = 0.660). In particular, the SVLT-DR score was significantly lower in the G(–)F(+) group compared to both the G(–)F(–) HC and LC groups (*P* < 0.001). Similarly, the MMSE and CDR-SOB scores showed the most pronounced decline in the focal uptake group [G(–)F(+)] compared to the G(–)F(–) HC and LC groups. However, there were no significant differences in the four cognitive measurement areas between the G(–)F(–) HC and G(–)F(–) LC groups. When comparing the G(–)F(+) and G(+) groups, the G(+) group exhibited significantly worse cognitive scores in SVLT-DR (*P* = 0.010), RCFT-DR (*P* = 0.031), and MMSE (*P* = 0.022).

### 3.4 Regional distribution of focal amyloid deposition

In the G(–)F(+) group, we analyzed the number of regions with amyloid deposition and their locations (Table 3). Out of the 82 participants, 71 (86.6%) had amyloid deposition confined to a single region, 9 participants (11.0%) showed deposition in two regions, and only 2 participants (2.4%) had deposition across three regions. None of the participants exhibited amyloid deposition in four or more regions. We observed a trend suggesting that an increasing number of focal amyloid depositions was associated with higher Centiloid values; however, this association did not reach statistical significance (*P* = 0.303). Also, in the comparison between participants with one focal region, there were no significant differences in the structural features and cognitive impairments.

**Table 3:** Demographics of the participants by region of focal amyloid deposition.

	**Focal one region (*****N*** **=** **71)**					**Focal two regions**	**Focal three regions**
	**Frontal**	**Temporal**	**Parietal**	**Cingulate**	**Striatum**		
No. (%)	1	34	17	11	8	9	2
Global Centiloid, mean ± SD	16.4	11.6 ± 5.5	15.5 ± 3.1	11.6 ± 6.3	0.2 ± 10.3	13.2 ± 4.9	18.4 ± 1.5
Age, mean ± SD, year	82.0	76.4 ± 6.7	74.2 ± 6.1	71.4 ± 6.8	77.0 ± 2.6	73.2 ± 7.2	70.5 ± 9.2
Female Sex, No (%)	1(100.0)	10(29.4)	13(76.5)	7(63.6)	0(0.0)	3(33.3)	1(50.0)
Education, mean ± SD, year	12.0	14.2 ± 4.0	11.3 ± 4.9	10.8 ± 4.3	14.8 ± 2.4	14.4 ± 3.8	14.0 ± 2.8
Diagnosis (CU/MCI), No.	0/1	11/23	7/10	8/3	4/4	1/8	0/2
APOE ε4 Carrier, No. (%)	0(0.0)	16(47.1)	10(58.8)	5(45.5)	1(12.5)	4(44.4)	0(0.0)
Hippocampal volumes (mm3)	4,926.375	5,406.955 ± 908.229	5,558.782 ± 922.287	5,614.412 ± 861.157	5,094.484 ± 513.212	5,277.447 ± 974.889	6,027.000 ± 772.515
Cortical Thickness (mm)	2.921	3.054 ± 0.106	3.073 ± 0.105	3.129 ± 0.106	3.024 ± 0.064	3.099 ± 0.128	3.079 ± 0.024
SVLT-DR (z-scores)	−1.860	−1.103 ± 1.278	−1.051 ± 1.318	−0.267 ± 1.321	−1.278 ± 0.733	−2.099 ± 0.863^‡^	−1.200 ± 0.000
RCFT-DR (z-scores)	−0.380	−0.768 ± 1.102	−1.165 ± 1.179	−0.312 ± 0.926	−1.413 ± 0.850	−1.668 ± 1.041	−1.135 ± 1.534
MMSE Score (z-scores)	−2.490	−0.898 ± 1.649	−0.667 ± 1.990	−0.532 ± 2.395	−1.469 ± 1.764	−2.377 ± 2.323	−1.145 ± 1.732
CDR-SOB	2.500	1.500 ± 1.777	2.094 ± 2.162	1.682 ± 2.016	2.688 ± 3.909	1.833 ± 1.299	1.500 ± 0.000

Unless otherwise indicated, data are expressed as mean ± standard deviation or *N* (%). SVLT-DR, Seoul verbal learning test-delayed recall; RCFT-DR, Rey-Osterrieth complex figure test-delayed recall; MMSE, mini-mental state examination; CDR-SOB, clinical dementia rating scale sum of boxes.

### 3.5 Sensitivity analysis

For sensitivity analysis (Table 4), we applied a cutoff value of 25.5 rdcCL obtained through GMM, and a threshold of 10 rdcCL so that cases classified as negative accurately reflected the absence of neuroinflammatory plaques (Amadoru et al., 2020). When using the 25.5 rdcCL cutoff, the rdcCL of the G(–)F(+) group (mean ± SD, 15.43 ± 7.83) had significantly higher values compared to the G(–)F(–) HC group (13.10 ± 3.62), which made direct comparison challenging. However, when comparing the G(–)F(+) and G(–)F(–) HC groups, the G(–)F(+) group showed significantly lower RCFT-DR (*P* < 0.001), MMSE score (*P* = 0.006), and higher CDR-SOB scores (*P* = 0.002).

**Table 4:** Comparison of the two groups for sensitivity test.

	**Global(–) Focal(+)**	**Global(–) Focal(-) High Centiloid**	***P* value**
**Sensitivity analysis with Centiloid Cut-off of 25.5**			
Global Centiloid	15.43 ± 7.83	13.10 ± 3.62	0.002
Hippocampal volumes (mm^3^)	5,423.02 ± 927.84	6,019.96 ± 865.84	0.109
Cortical Thickness (mm)	3.08 ± 0.12	3.14 ± 0.10	0.085
SVLT-DR (z-scores)	−0.96 ± 1.33	−0.57 ± 1.29	0.176
RCFT-DR (z-scores)	−0.85 ± 1.18	−0.25 ± 1.17	< 0.001
MMSE Score (z-scores)	−0.99 ± 1.87	−0.38 ± 1.27	0.006
CDR-SOB	1.74 ± 2.09	1.03 ± 0.82	0.002
**Sensitivity analysis with a Centiloid Cut-off of 10**			
Global Centiloid	2.39 ± 7.17	7.34 ± 1.40	0.005
Hippocampal volumes (mm^3^)	5,480.51 ± 755.08	6,091.58 ± 910.39	0.658
Cortical Thickness (mm)	3.04 ± 0.10	3.12 ± 0.10	0.809
SVLT-DR (z-scores)	−1.17 ± 1.12	−0.45 ± 1.22	0.156
RCFT-DR (z-scores)	−0.84 ± 1.04	−0.30 ± 1.15	0.089
MMSE Score (z-scores)	−1.12 ± 1.55	−0.24 ± 1.23	0.012
CDR-SOB	1.93 ± 2.50	0.87 ± 0.71	< 0.001

Values are presented as the mean ± standard deviation. We applied a cutoff value of 25.5 rdcCL and a threshold of 10 rdcCL for the sensitivity analysis. Statistical analysis was performed using analysis of covariance (ANCOVA). Covariates used for neuropsychological test scores included age, sex, *APOE* ε4, and education level, whereas age, sex, *APOE* ε4, and ICV were used for the analysis of structural differences. SVLT-DR, Seoul Verbal Learning Test-delayed recall; RCFT-DR, Rey-Osterrieth Complex figure Test-delayed recall; MMSE, Mini-Mental State Examination; CDR-SOB, Clinical Dementia Rating Scale Sum of Boxes.

When applying a cutoff of 10 rdcCL and dividing the groups into quintiles, the G(–)F(–) HC group (7.34 ± 1.40) exhibited significantly higher Centiloid values compared to the G(–)F(+) group (2.39 ± 7.17; *P* = 0.005). However, the G(–)F(+) group demonstrated significantly lower scores on the MMSE (*P* = 0.012) assessments, as well as a higher score on the CDR-SOB *(P* < 0.001) in comparison to the G(–)F(–) HC group.

## 4 Discussion

In this study, we aimed to identify factors associated with cognitive impairment and structural abnormalities in a subgroup of patients with subthreshold amyloid burden among individuals without dementia (CU and MCI). The key findings of this study included the identification of structural changes and cognitive impairment in participants with focal amyloid deposition. Further, our results suggest that focal amyloid measurement using imaging is an effective classification method for patients with focal amyloid accumulation. Finally, it was found that approximately 3% of the total participants exhibited focal uptake, despite being globally negative. These results highlight the cognitive impairment in patients with focal amyloid deposits, highlight the importance of accurate amyloid measurement methods, and emphasize the importance of considering patients with focal amyloid uptake in dementia research and management.

The first major finding of this study was that patients with focal amyloid uptake in the group with subthreshold levels of amyloid showed significant differences as compared to the group of globally negative patients with high CL levels. Previous research has consistently demonstrated structural and neuropsychological changes in the focal amyloid uptake group compared to the globally negative group. In addition to these findings, when comparing the focal amyloid uptake group [G(–)F(+)] with a group exhibiting similar levels of amyloid but without focal uptake [G(–)F(–) HC], we observed more pronounced brain structural changes and lower cognitive performance in the focal uptake group. Moreover, the G(–)F(+) group included a higher proportion of patients with MCI than the G(–)F(–) HC group. These results provide evidence supporting the hypothesis that the occurrence of structural abnormalities and cognitive impairment in G(–)F(+) group is influenced by focal amyloid uptake, leading to additional amyloid accumulation and clinical progression, rather than being solely driven by elevated amyloid levels.

In sensitivity analyses using the originally derived threshold of 25.5 CL, as well as the threshold of 10 CL, statistically significant differences in CL values were observed between the G(–)F(+) and G(–)F(–)HC groups, making direct comparisons challenging in both sensitivity analyses. However, even with a threshold of 25.5 rdcCL, patients with focal amyloid uptake still demonstrated significant structural deterioration and cognitive impairment compared with the G(–)F(–)HC group. In addition, when compared using a rather low threshold of 10 rdcCL, the G(–)F(+) group also demonstrated lower hippocampal volume, cortical thickness, and SVLT-DR scores than the G(–)F(–)HC group. These results provide further evidence to support the hypotheses of this study.

The second major finding of this study pertains to the accuracy of regional amyloid measurements and the reliability of the threshold values. The conventional SUVR method demonstrated a high correlation between the FBB and FMM ligands (Cho et al., 2020b); however, significant differences in SUVR values were observed across multiple regions. Therefore, previous studies required the use of different thresholds for each ligand to differentiate between positive and negative cases (Kim H. R. et al., 2021). In contrast, in the present study, we used the MRI-regional CL obtained from a previous study (Kim et al., 2022), allowing the standardization of the two ligands. This technique facilitated comparisons and streamlined numerical assessments, even with multiple ligands, leading to advantages in terms of ease of comparison and numerical simplicity. The threshold values maintained a similar level to those reported in previous studies, while the application of the rdcCL 25.5 threshold for dividing global CL exhibited a visually discernible value consistent with previous literature (Amadoru et al., 2020; Collij et al., 2021).

In this study, the visual assessment of patients with focal uptake showed that only 14.9% tested as positive; conversely, when considering the entire patient population, a high level of accuracy (92.1%) was observed. This suggests that the conventional method of visual assessment aids in detecting early amyloid pathology, but may be helpful in identifying the early progression of amyloid at subthreshold levels by utilizing CL and threshold-based detection of focal uptake.

As the final major finding, we observed that approximately 3% of the total patient cohort exhibited focal amyloid uptake, despite being globally negative. Although this ratio was lower than that found in previous studies (Kim et al., 2020; Kim S. E. et al., 2021; Kim et al., 2023), it was comparable to the ratio of 13.3% reported in a preliminary study performed prior to this research. These findings suggest that, while the proportion of focal participants within the overall and negative populations decreases as the sample size increases, it remains largely at a consistent level without extreme reduction. From this perspective, we confirmed that the amyloid focal deposition group generally exists within the global negative group. Overall, these findings suggest that individuals with focal amyloid deposition, despite being classified as negative, are more likely to have cognitive impairment, and that a certain proportion of such patients exist within the amyloid-negative group. Based on these findings on associated factors, clinicians and researchers may wish to further investigate the factors and specificity that contribute to the high rate of cognitive impairment and structural changes in these focal-positive patients, which could provide a deeper understanding of the association between amyloid and dementia.

Nevertheless, there were some limitations to this study. First, instead of the original threshold of 25.5 rdcCL obtained through GMM, we had to use the Klunk Centiloid threshold of 20 rdcCL for moderate plaque density, as reported in another paper (Amadoru et al., 2020), This change was necessary because a statistically significant difference in rdcCL values was observed between the G(–)F(+) and G(–)F(–)HC groups, which made direct comparisons difficult. Second, to establish conclusive evidence for the AD progression, longitudinal studies comparing the amyloid accumulation in patients showing focal uptake with other groups over an extended period were not conducted. Further longitudinal research is therefore needed to investigate the differences between these groups. Third, rdcCL from our new methods is not the same as Klunk CL. However, this argument might be mitigated by our findings that rdcCL were very highly correlated with Klunk's Centiloid values (*R*^2^ = 0.995) (Supplementary Figure S1). Finally, in the original design, it was expected that patients with low amyloid levels would show relatively mild cognitive impairment and lesser structural changes, indicating a normal state. However, in the present study, participants with low amyloid levels [G(–)F(–)LC] did not significantly differ from the G(–)F(–)HC group in RCFT-DR and hippocampal volume, but rather had lower cortical thickness. Overall, the low CL group appeared to be older, and their ratio of CU to MCI (CU/MCI) paralleled that of the high CL group, implying that these changes may be attributable to factors beyond amyloid deposition, such as SNAP (Jack et al., 2016). In particular, cerebrovascular factors might play a role, such as diabetes mellitus. Despite these limitations, our study included both FMM and FBB ligands, integrating them through rdcCL, to achieve a larger sample size compared to previous studies, and demonstrated significant progression compared to patients with similar amyloid levels.

## 5 Conclusions

Overall, we found that patients with subthreshold focal amyloid uptake had statistically more cognitive impairment and structural changes than patients with similar amyloid levels without focal uptake. These findings suggest that focal amyloid deposition may contribute to the development of cognitive impairment and structural changes. These results indicate that clinicians should monitor patients with focal uptake, even those with low amyloid levels, as they may benefit from early interventions.

## Data Availability

The raw data supporting the conclusions of this article will be made available by the authors, without undue reservation.
