# Supplementary figures and images for: Association between focal amyloid deposition and cognitive impairment in individuals below the amyloid threshold
Source: Front Aging Neurosci. 2024 Oct 30;16:1452081. doi: 10.3389/fnagi.2024.1452081 (PMC11557402; doi:10.3389/fnagi.2024.1452081)

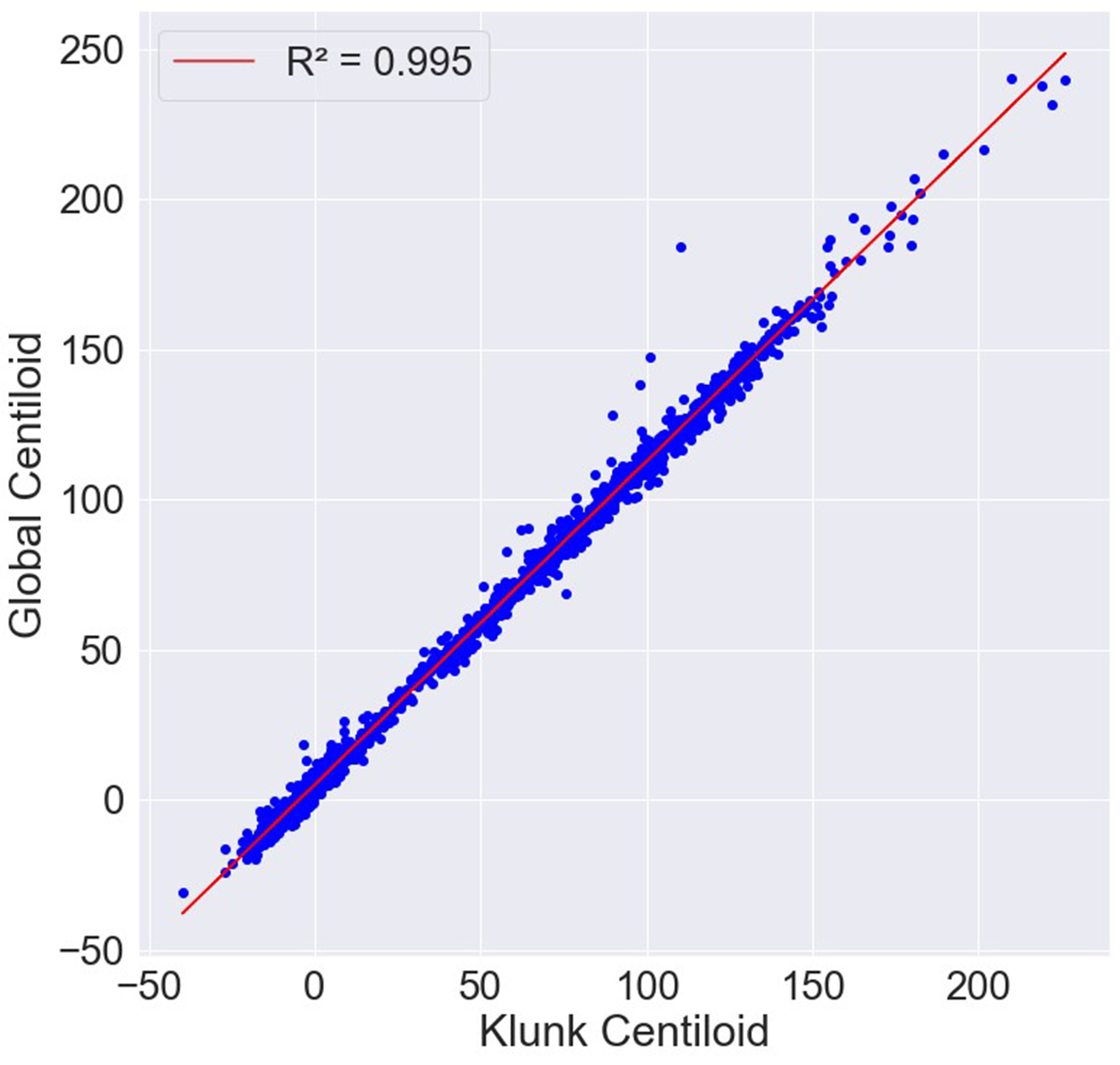

Supplement: Supplementary file 1 [file Image_1.JPEG]
